# Metal Oxide Nanomaterial QNAR Models: Available Structural Descriptors and Understanding of Toxicity Mechanisms

**DOI:** 10.3390/nano5041620

**Published:** 2015-10-12

**Authors:** Jiali Ying, Ting Zhang, Meng Tang

**Affiliations:** 1 Key Laboratory of Environmental Medicine Engineering, Ministry of Education, School of Public Health, Southeast University, Nanjing 210009, China; E-Mail: yingjiali1020@163.com; 2 Jiangsu key Laboratory for Biomaterials and Devices, Southeast University, Nanjing 210009, China

**Keywords:** nanotoxicology, metal oxide, quantitative structure activity relationship (QSAR), descriptor, toxicity mechanisms

## Abstract

Metal oxide nanomaterials are widely used in various areas; however, the divergent published toxicology data makes it difficult to determine whether there is a risk associated with exposure to metal oxide nanomaterials. The application of quantitative structure activity relationship (QSAR) modeling in metal oxide nanomaterials toxicity studies can reduce the need for time-consuming and resource-intensive nanotoxicity tests. The nanostructure and inorganic composition of metal oxide nanomaterials makes this approach different from classical QSAR study; this review lists and classifies some structural descriptors, such as size, cation charge, and band gap energy, in recent metal oxide nanomaterials quantitative nanostructure activity relationship (QNAR) studies and discusses the mechanism of metal oxide nanomaterials toxicity based on these descriptors and traditional nanotoxicity tests.

## 1. Introduction

Nanotechnology involves the study of the synthesis, characterization, and properties of nanomaterials [1]. The emergence of nanotechnology has led to innovations in many areas of electronics, energy management, structural materials, functional surfaces, construction, information technology, pharmaceuticals, and medical devices. The largest share of the manufacturing and application market among the different nanomaterials belongs to metal oxide nanomaterials, they are already used in some consumer products, such as TiO_2_ in paints and ZnO in sunscreen products [2]. Moreover, since the 1950s, millions of tons of materials such as carbon black, SiO_2_, TiO_2_, and Al_2_O_3_ have been produced per year for use in paints, catalysts, and surfactants [3]. Nanomaterials can release into the environment during production, transport, and use [4], and metal oxide nanomaterials are no exception [5]. The divergent published toxicology data on metal oxide nanomaterials makes it difficult to determine whether there is a risk associated with metal oxide nanomaterial exposure [2]. The small size and large specific surface area of metal oxide nanomaterials endows them with high chemical reactivity and intrinsic toxicity [6]. Therefore, most nanotoxicity studies have concentrated on metal oxide nanomaterials, and different metal oxide nanomaterials have different toxic effects according to previous studies [7,8,9,10,11,12,13,14,15,16,17].

However, performing toxicology tests for each metal oxide nanomaterial is time consuming and resource intensive; therefore, researchers are developing computational nanotoxicology methods, such as quantitative structure activity relationship (QSAR) modeling, to predict the toxicity of metal oxide nanomaterials. Such predictions would allow researchers to prioritize toxicology tests on real metal oxide nanomaterials [18]. QSAR modeling was originally a computational chemistry method; it is known as nano quantitative structure activity relationship (nano-QSAR) [19] or quantitative nanostructure activity relationship (QNAR) [20] modeling in nanotoxicology. In this review, we use the term QNAR modeling. QSAR modeling attempts to correlate a set of structural parameters (which are called molecular descriptors) that describe a chemical compound with its biological activity. The QSAR modeling procedure includes the selection of molecular descriptors and toxicology endpoint descriptors [21,22], as well as construction, validation [23,24,25], and evaluation [19] of the model. It is not our purpose to discuss every procedure in QNAR modeling here, which have been reviewed by many other researchers [3,19,20,22,26,27,28,29,30]. Current reviews about QNAR research in the past five years have been listed in Table 1. Instead, we focus on the molecular descriptors in this review because, as we know, we use metal oxide nanomaterial QNAR method to high-throughput screening of the toxicity of metal oxide nanomaterials and to guide the production of metal oxide nanomaterials, the most important steps to improve QNAR models are the selection of structural descriptors, to help readers moving forward in the implementation of QNAR, we listed the available structural descriptors and further analyzed the key issues in metal oxide QNAR study, and to the best of our knowledge, they have not been reviewed before.

**Table 1:** Quantitative nanostructure activity relationship (QNAR) reviews in the past five years.

Title	Summary	Ref
No time to lose—high throughput screening to assess nanomaterial safety	This review aims to provide a comprehensive introduction to the high throughput/content screening methodology employed for safety assessment of engineered nanomaterials, including data analysis and prediction of potentially hazardous material properties.	[3]
Exploring QNAR modeling as a tool for predicting biological effects of manufactured nanoparticles	The review discusses major approaches for model building and validation using both experimental and computed properties of nanomaterials by considering two different categories of nanomaterials datasets:(i) those comprising nanomaterials with diverse metal cores and organic decorations;(ii) those involving nanomaterials possessing the same core.	[20]
Predictive models for nanotoxicology: Current challenges and future opportunities	The review aims to provide researchers strategies for directing research towards predictive models and the ancillary benefits of such research.	[19]
Applying quantitative structure-activity relationship approaches to nanotoxicology: Current status and future potential	The purpose of this review is to provide a summary of recent key advances in the field of QNAR modelling, to identify the major gaps in research required to accelerate the use of QSAR methods, and to provide a road map for future research needed to achieve QSAR models useful for regulatory purposes.	[26]
Advancing risk assessment of engineered nanomaterials: Application of computational approaches	The purpose of this review is to present the current state of knowledge related to the risks of the engineered nanoparticles and to assess the potential of efficient expansion and development of new approaches, which are offered by application of theoretical and computational methods, applicable for evaluation of nanomaterials.	[27]
Nano(Q)SAR: Challenges, pitfalls and perspectives	This article aims to identify some of the pitfalls and challenges associated with (Q)NARs. Three major barriers were identified: the need to improve quality of experimental data in which the models are developed from, the need to have practical guidelines for the development of the (Q)NAR models and the need to standardise and harmonise activities for the purpose of regulation.	[28]

In classical QSAR modeling, these molecular descriptors typically relate to the steric and electronic properties of the chemical compound and can be measured experimentally or computationally. Classical QSAR studies usually begin from the stage of molecular modeling and descriptor calculation [31,32]. There are many ways to classify classical molecular descriptors, such as theoretical molecular descriptors include 0D-descriptors, 1D-descriptors, 2D-descriptors, 3D-descriptors, and 4D-descriptors [33]. However, the molecular descriptor of a nanomaterial is different from classical QSAR molecular descriptors, and we should give it a proper name “structural descriptor” in QNAR modeling because nanomaterials are no longer simple chemical compounds. The metal oxide nanomaterials QSAR study should be classified by two categories, (i) coated metal oxide nanomaterials QSAR study: the selection of coated metal oxide nanomaterials structural descriptors is always the structural descriptor of its organic surface modification; and (ii) bare metal oxide nanomaterials QSAR study: the selection of bare metal oxide nanomaterials structural descriptors is a challenge in metal oxide QNAR model-building because of its inorganic composition and nanostructure. For the first type of study, the surface modification of coated metal nanomaterials was considered to be the key factor to influence its toxicity [34,35]; therefore, we can refer to it as organic chemicals QSAR study. However, the relationship between the surface modification group of metal oxide nanomaterials and nanotoxicity is not the focus of this article. In this review, we emphasize on bare metal oxide nanomaterials QSAR study; all we discuss below is about bare metal oxide nanomaterials. Based on previous studies [36,37,38,39,40,41], we have classified the metal oxide nanomaterial structural descriptors as experimental descriptors (describing the morphological structural properties and physicochemical properties of metal oxide nanomaterials) and theoretical descriptors (describing the constitutional properties, electronic or thermodynamic properties); in addition, we summarize how to obtain these structural descriptors and introduce some novel structural descriptors used in existing metal oxide QNAR models. Finally, and also drawing on recent nanotoxicity studies, we propose mechanisms for metal oxide nanomaterial toxicity from the existing QNAR models based on these descriptors. We hope that this article will be useful for researchers, especially for toxicologists, in performing metal oxide nanomaterial QNAR research.

## 2. Experimental Descriptors

Metal oxide nanomaterial experimental descriptors are usually experimental measurements, such as size distribution, agglomeration state, shape, porosity, surface area, and chemical composition. Metal oxide nanomaterials exhibit different properties than bulk materials or isolated molecules. Knowing the morphology and physicochemical properties of metal oxide nanomaterials can open new exciting opportunities for modeling metal oxide nanomaterial characteristics through the use of the QSAR approach, so it is meaningful to classify these experimental measurements based on morphological structural properties and physicochemical properties which provide researches different views to select structural descriptors. Here we use size, shape, porosity, and surface area as morphological structural properties in addition to zeta potential and surface modification [6,7,16,42,43].

### 2.1. Morphological Structural Properties

Metal oxide nanomaterials can be characterized by different sizes, shapes, porosities, surface areas, crystals, and aggregation; these characteristics are usually classified as physico-chemical properties, and we classify them as morphological structural properties in this review. Many techniques are complementary and used together to support the data. Various techniques such as scanning electron microscopy (SEM), transmission electron microscopy (TEM), dynamic light scattering (DLS), static light scattering (SLS), and atomic force microscopy (AFM) are available for primary size, size distribution, agglomeration state, porosity, and shape characterization [42,43,44,45]. Brunauer-Emmett-Teller (BET) surface area measurements can be used to measure a given amount of metal oxide nanomaterial based on the gas adsorption surface area, as well as the porosity of the samples [44,46,47,48,49]. X-ray diffraction (XRD) analysis is a classical crystallographic structure measurement technique that can be used for metal oxide nanomaterials with respect to the arrangements of atoms in the bulk as well as on the surface. This technique uses the patterns, positions, intensities, and shapes of the peaks to elicit the atomic structure of the samples. Moreover, small angle X-ray scattering (SAXS) can also be used to reveal the crystallographic properties of metal oxide nanoparticles [50,51,52]. Gajewicz [40] calculated 11 image descriptors (area (*A*), volume (*V*), surface diameter (*Ds*), volume/mass diameter (d*V*/*m*), volume/surface diameter (dSauter), aspect ratio *x* (AR_*x*), aspect ratio *y* (AR_*y*), porosity *X* (P*X*), porosity *Y* (P*Y*), sphericity (Ψ), and circularity (fcirc)) based on TEM microscopic images, which can reflect the size, size distribution, shape, porosity, and surface area for all 18 nanometer-sized metal oxides. These descriptors were not included in the final model; however, they provided us with a method to quantify the image characterization. Liu *et al*. [53] used nanoparticle primary size and volume fraction (in solution) as two of the structural descriptors to build a classification nano-SAR model, and the classification accuracy for both internal validation and external validation was 100%. Size has also been used in metal oxide nanomaterials QNAR models [54,55,56,57,58].

### 2.2. Physicochemical Properties

Physicochemical properties include zeta potential, acidity coefficient (pK_a_), relaxivities, isoelectric point, and surface charge. The zeta potential of nanomaterials is measured by light-scattering electrophoresis or electro-acoustophoresis, and the isoelectric point (IEP) can be determined using electrophoretic mobility measurements (EPM). pK_a_ can be measured by titration in various suspending media. Liquid contact angle assessment reveals additional information about surface chemistry, charge, and energy. Elemental speciation and redox state can be measured by Raman, X-ray absorption fine structure (XAFS), X-ray absorption near edge structure (XANES), and nuclear magnetic resonance (NMR), and electronic, magnetic, and photonic properties can be analyzed by small- and wide-angle X-ray scattering (SAXS), Mossbauer, electron spin resonance (ESR), Raman, and ultraviolet-visible spectroscopy (UV-Vis). “Dustiness” or tendency to aerosolize, can be measured by a scanning mobility particle sizer (SMPS). Charge density, pK_a_, point of zero charge (PZC), and ionization fraction can be tested by direct titration in various suspending media [3]. The total concentration of the metal oxide can be measured by X-ray fluorescence (XRF), and the concentration of soluble metal can be measured by inductively coupled plasma mass spectrometry (ICP–MS) conjugation with ultrafiltration [59]. Band gap energy (Eg) can be measured by UV-Vis spectroscopy [60]. UV-Vis spectroscopy can also be used as an alternative method to roughly evaluate nanoparticle sedimentation. Redox potential can be measured using an oxidation reduction potential (ORP) electrode probe [61]. Duffin *et al*. [62] proposed approaches that presented the possibility of modeling the potential toxicity of metal nanoparticles and nuisance dusts based on the inflammatory response under an instilled surface area dose. The zeta potential and IEP have been included in a metal oxide nanomaterial nanoSAR model [53]. Singh *et al*. [56] used relativities (*R*_1_, *R*_2_) and zeta potential as four of the model’s parameters to predict the toxicity of 51 different NMs (with diverse metal cores) in four cell lines. Fourches used the zeta potential and relativities to build a nanoQSAR model [58]. Cho *et al*. [63] correlated the percentage of granulocytes of rats exposed to metal oxide nanomaterials with the zeta potential in physiological saline (pH 5.6) of these metal oxide nanomaterials. Main experimental structural descriptors used in some metal oxide QNAR models was reviewed in Table 2.

**Table 2:** Main experimental structural descriptors used in some metal oxide (Q)NAR models.

Experimental structural descriptors						Ref
Size	Volume fraction	Zeta potential	Relativities *R*_1_	Relativities *R*_2_	IEP	
√	√	√			√	Liu *et al*. [53]
		√	√	√		Epa *et al*. [54]
		√	√	√		Singh *et al*. [56]
		√	√	√		Fourches *et al*. [58]
		√				Cho *et al*. [63]

## 3. Theoretical Descriptors

Theoretical descriptors usually involve applications of quantum chemical or molecular simulation methods that have been proven to be reliable and efficient means to predict molecular properties. Classical theoretical molecular descriptors are derived from a symbolic representation of the molecule and can be further classified according to the different types of molecular representation. Similar to classical molecular descriptors, theoretical descriptors are an important part of metal oxide nanomaterials QSAR study, but with their own special properties; metal oxide nanomaterials consist of metal oxide crystal structures, and we should, thus, first determine their crystal structure.

### 3.1. Constitutional Properties

Constitutional properties are simple molecular descriptors that can be obtained from the periodic table of elements, chemical handbooks, and molecular formulae, such as the molecular weight of the molecule, weight of the atom, number of molecular descriptors, and cation charge. This is a cost-effective approach to obtain molecular descriptors without depending on quantum software. Hu *et al*. [6] reported that the cytotoxicity of seven metal oxide nanoparticles was observed to be correlated with their cation charges. Kar *et al*. [39] used metal electronegativity (χ), the charge of the metal cation corresponding to a given oxide (χOx), atomic number and valence electron number of the metal as simple molecular descriptors to develop QSAR models for predicting the cytotoxicity of 17 metal oxide nanomaterials to *Escherichia coli*. Moreover, the period of the nanoparticle metal has also been used in a metal oxide nanomaterials cytotoxicity classification model [53].

### 3.2. Electronic Properties

Electronic properties are properties relate to energy and are the most widely used properties in metal oxide QNAR model building. Some electronic properties of metal oxides can be used as descriptors, such as band gap and valence gap energy. However, most metal oxide nanomaterials are difficult to dissolve in solution, they are nano-size in water; so the unusual properties of nanoparticles, such as their hardness, high yield strength, flexibility, rigidity, and ductility, are related to their high surface-to-volume ratio, and the quantum size effect and macro-quantum tunneling effect are attributable to their nano-size [64]. Therefore, it is necessary to determine the metal oxide properties on the nanoscale. Fortunately, some contributions have highlighted that the most significant size-dependent changes of some properties of nanoparticles are observed below approximately 5 nm; thus, changes in nanomaterials properties between 15 and 90 nm can be ignored [65,66]. Burello *et al*. [67] proposed a theoretical framework of a descriptor calculation that is related to the oxidative stress potential of oxide nanoparticles based on the assumption that a particle has a diameter larger than 20–30 nm and no surface states in the band gap. Since some quantum-chemical properties change with the diameter of the nanoparticle [68], another approach involves software-based calculations (e.g., CODESSA [69], DRAGON [70], CAChe [71], and MOE [72]). However, from a quantum chemistry viewpoint, nanoparticles (10–1000 Å) are very large systems [68]; therefore, it is necessary to maximally simplify the molecular models used for geometry optimization and/or calculation of the molecular properties to reflect the variability of the nanoparticles' structures [19]. Gajewicz *et al*. [68] observed that molecular properties change with size in two ways based on MOPAC software; one way is that the parameters vary non-linearly with the considered crystal size (e.g., ZnO, 11 Å) until they reach a saturation point (type A), and the other way is that the parameters vary linearly with the crystal size. Therefore, we can use the saturation point to determine the type A properties and use the linear equation to determine the type B properties with calculations based on many atoms.

Puzyn *et al*. [41] used the ΔH_Me+_ descriptor that represents the enthalpy of formation of a gaseous cation with the same oxidation state as that in the metal oxide structure as a parameter to construct the toxicity prediction model for *Escherichia coli*. Gajewicz *et al.* [40] combined experimental–theoretical studies to develop an interpretative QNAR model to predict the toxicity of 18 nano-metal oxides to the HaCaT cell line. A simple and statistically significant QSAR model was successfully developed for the dark group based on two descriptors, the absolute electronegativity of the metal and of the metal oxide. Similarly, the best correlation was obtained to predict the photo-induced toxicity of metal oxide nanomaterials using two descriptors, the molar heat capacity and average of the alpha and beta lowest unoccupied molecular orbital (LUMO) energies of the metal oxide [37]. Liu *et al*. [73] used the atomization energy of the metal oxide, atomic mass of metal nanoparticles, conduction band energy of the nanoparticles, metal oxide ionization energy, and metal oxide electronegativity as descriptors to construct several metal oxide nanomaterial toxicity classification models. Main theoretical structural descriptors used in some metal oxide QNAR models are reviewed in Table 3.

**Table 3:** Main theoretical structural descriptors used in some metal oxide QNAR models.

Structural descriptors	Ref
Cation charges	Hu *et al*. [6]
The absolute electronegativity of the metal and of the metal oxide, the molar heat capacity and average of the alpha and beta LUMO	Pathakoti *et al*. [37]
Metal electronegativity, the charge of the metal cation, atomic number, valence electron number of the metal	Kar *et al*. [39]
Standard enthalpy of formation of metal oxide nanocluster, Mulliken’s electronegativity	Gajewicz *et al*. [40]
The enthalpy of formation of a gaseous cation with the same oxidation state as that in the metal-oxide structure	Puzyn *et al*. [41]

## 4. Other Novel Descriptors

Actually, except these structural descriptors listed above, some new methods appeared to select more comprehensive structural descriptors for researchers.

### 4.1. Liquid Drop Model

The other novel descriptor is the “liquid drop” model (LDM). The LDM was applied to describe the supramolecular structure of nanoparticles [36]. It is beneficial to use a combination of descriptors that reflect the structure of the nanoparticles for the different levels of organization: from a single molecule to a supramolecular ensemble of molecules, to the interactions of nanomaterials with biological systems. Sizochenko *et al*. [36] established an LDM for 17 metal oxide nanomaterials to predict the toxicity toward *Escherichia coli* and HaCaT cells.

### 4.2. QSAR-Perturbation Approach Based Descriptors

Luan *et al*. [38] used the descriptor DDV (*ca*.) to characterize the physicochemical properties, as well as the types of cells against which the metal oxide nanomaterials were tested. The descriptors represent their relative degrees of importance in the QSAR-perturbation model. This model can simultaneously predict the cytotoxicity of different nanoparticles against several mammalian cell lines while considering different times of exposure of the cell lines, as well as the chemical composition of the nanoparticles, their size, the conditions under which the size was measured, and their shape.

### 4.3. Optimal SMILE-Based Descriptor

SMILES is a sequence of symbols which represent the molecular architecture. It has been applied in QSAR study of carbon nanotubes [74] and some organic chemicals [75]. It has been proven it can be applied to metal oxide nanomaterials QNAR model building. Toropova *et al*. [76] constructed a model to predict the membrane damage caused by a group of zinc oxide and titanium oxide nanomaterials; Toropova *et al*. [77] built up a model for prediction of dark cytotoxicity and photo-induced cytotoxicity of metal oxide nanoparticles to the bacteria *Escherichia coli*. The advantages and disadvantages of different structural descriptors in metal oxide QNAR studies can be seen in Table 4.

**Table 4:** Advantages and disadvantages of different structural descriptor types in metal oxide QNAR studies.

Descriptors type	Advantages	Disadvantages
morphological structural properties	directly relate to the characteristics of metal oxide nanomaterials, easy to explain the toxicity mechanism	measuring error, some of the properties are difficult to quantitate
physicochemical properties	directly relate to characteristics of metal oxide nanomaterials, easy to explain the toxicity mechanism	measuring error
constitutional properties	easy to obtain	characteristics of metal oxide nanomaterial are not included
electronic properties or thermodynamic properties	easy to obtain , easy to explain the toxicity mechanism	the calculation system is relatively small
novel descriptors	directly relate to the characteristics of metal oxide nanomaterials, easy to explain the toxicity mechanism	the calculation method is complex

## 5. Understanding Toxicity Mechanism(s) from Existing QNAR Models

In traditional nanotoxicity studies, the toxicity of metal oxide nanomaterials is related to their size, size distribution, surface area, shape, zeta potential, surface charge, aggregation state, and the extent of ion detachment from the surface [2]. Karlsson *et al*. [10] discovered that nano-sized copper oxide was much more toxic than micro-sized copper oxide toward A549 cells. Hamilton *et al*. [78] observed that the alteration of anatase titanium dioxide nanomaterials into a fiber structure of greater than 15 μm was highly toxic and initiated an inflammatory response by alveolar macrophages. Fröhlich *et al*. [79] demonstrated that charge density and hydrophobicity are equally important in non-phagocytic cell ingestion, but that the cellular uptake of cationic NPs is higher than that of anionic NPs in these cells; phagocytic cells, however, preferentially take up anionic nanomaterials. Rogers [80] observed that body length and progeny count decreased and organismal stress increased in *Caenorhabditis elegans* when exposed to aggregated cerium oxide (0–17.21 ug/mL). Heinlaan *et al*. [5] noticed that the metal ions detached from the surface were responsible for the toxicity of nano-sized zinc oxide and nano-sized copper oxide in *Vibrio fisheri* bacteria. However, in recent metal nano-QSAR studies, size, size distribution, shape, surface area, zeta potential, surface charge, and aggregation state were not the key factors that affected the toxicity of the metal oxide nanomaterials, and some descriptors related to the release of ions detached from the metal oxide were often included in the models. Puzyn *et al*. [41] observed that the particle size of the metal oxide nanomaterials did not affect the antibacterial activity in their model; however, the descriptor that represented the enthalpy of formation of the gaseous cation having the same oxidation state as that in the metal oxide structure was used to build a satisfactory model that can be applied to predict the EC50 of *E. coli*. Kar *et al*. [39] used some simple molecular descriptors, such as metal electronegativity, charge of the metal cation corresponding to a given oxide, atomic number and valence electron number of the metal, to develop QSAR models for the EC50 of metal oxide nanoparticles toward *E. coli*. Pathakoti *et al*. [37] used the absolute electronegativity of the metal atom and the absolute electronegativity of the metal oxide to predict EC50 of metal oxide nanomaterials to *E. coli* in dark conditions, and used the literature molar heat capacity of the metal oxide at 298.15 K and the average of the alpha and beta lowest unoccupied molecular orbital energy of the metal oxide to predict the photo-induced toxicity of the metal oxide nanomaterials. Gajewicz *et al*. [40] used two descriptors (the enthalpy of formation of the metal oxide nanocluster representing a fragment of the surface and the Mulliken’s electronegativity of the cluster) to predict the median lethal concentration (LC50) of metal oxide nanomaterials toward HaCaT cells. The primary size was only included in a few metal oxide toxicity classification nanoSAR models [53,73].

We proposed a possible toxicity mechanism of metal oxide nanomaterials to explain these conflicts. Size, size distribution, shape, surface area, zeta potential, surface charge, and aggregation state are not direct factors that cause cell inhibition, cell death, or cell apoptosis. They can affect the cellular uptake of metal oxide nanomaterials by van der Waals forces, steric interactions, and electrostatic charges [3]. It can be performed by directly affecting the function of the cell membrane [81,82], or indirectly performed by some chemical activities, such as influencing the formation of protein corona [83], than finally influence their ability to enter cell. After entering the cell, metal oxide stability is an important factor that causes toxic effects, such as ions detaching from the surface of metal oxide nanomaterials, inducing ROS generation and then causing a series of oxidative stress reactions related to cell viability, cell apoptosis and cell death. In addition, we cannot ignore extracellular ROS and ions release induced toxicity [83,84]. From the characterization aspect, surface charge is usually reported as the zeta potential, which takes into account the electric potential in the interfacial double layer as well as the pK_a_ of the particle [85]. The surface charge of nanomaterials is particularly interesting as it is one of the factors that controls the dispersion and aggregation of engineered nanomaterials as well as affecting cellular uptake [86]. Surface charge may determine binding sites for receptors, affect the dispersion and aggregation of the particles, and affect the capacity to produce reactive oxygen species (ROS) [79]. Stable metal oxides do not exhibit any toxic effects, whereas metallic nanomaterials that have a redox potential may be cytotoxic and genotoxic [87]. The stability is found to be of prime importance to dictate ZnO QDs toxicity either towards the ROS generation or towards the liberation of metal [88]. Heavy metal ions induce oxidative stress and inflammatory responses and electron-hole pair generation during photoactivation, leading to free-radical generation [3], which is consistent with the results from some metal oxide nanomaterials QNAR studies.

**Figure 1:**
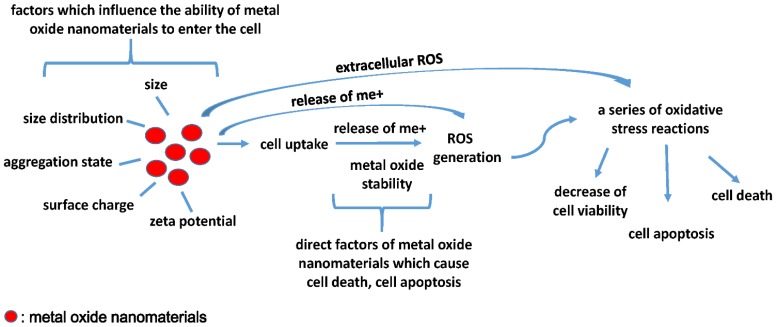
Characteristics such as size, size distribution, aggregation state, surface charge, and zeta potential can affect the cellular uptake of metal oxide nanomaterials. After entering the cell, metal oxide stability is an important factor that causes toxic effects, such as ions detaching from the surface of metal oxide nanomaterials, inducing ROS generation and then causing a series of oxidative stress reactions related to cell viability, cell apoptosis and cell death. In addition, extracellular ROS and released ions can also induce a series of oxidative stress reactions.

In a 2008 NATO workshop, experts proposed that toxicity may occur via one or a combination of four possible mechanisms [89]. The first mechanism suggested that the release of the chemical constituents from the nanomaterials leads to toxicity, such as the release of toxic anions. The second mechanism suggested that the size and shape of the particle produces steric hindrances or interferes with some important binding sites. The third mechanism of toxicity is related to the surface properties of the materials, such as local electric fields, electronic semi-conductance and charge densities. Finally, the fourth mechanism of toxicity involves the capacity of nanomaterials to act as vectors for transporting other toxic chemicals to sensitive tissues. The results of the QNAR study and nanotoxicity test study are consistent with these mechanisms. We combined metal oxide nanoparticle toxicity mechanisms with a recent metal oxide nanomaterials QNAR study that included a toxicity test, as shown in Figure 1.

## 6. Conclusions and Outlook

Metal oxide nanomaterials QNAR modeling is a comprehensive task that requires the combined efforts of toxicologists, inorganic chemists, materials scientists, and statisticians. We aim to provide readers available structural descriptors and the method to obtain these structural descriptors after reading this review. Recent studies have demonstrated that QNAR modeling is an effective and accurate tool to assess the biological activity and toxic potential of metal oxide nanomaterials in a short period with reduced cost. On one side, we use QNAR model to predict the toxicity of metal oxide nanomaterials; however, there were unavoidable problems in recent QNAR models, which are discussed here. First, most of the recent models were based on *in vitro* toxicity tests; there are few metal oxide nanomaterials QNAR models based on *in vivo* toxicity tests or model organisms, even though the toxicity data should involve multiple species in different environmental systems. Unavoidably, it was difficult to obtain some quantitative toxicity data for these organisms, such as median lethal dose (LD50) and lethal concentration 50 (LC50), because the toxicity of some metal oxide nanomaterials was very low. Second, the elucidation of the mechanism of toxicity appears to be a complex problem, with many studies reporting conflicting results. The toxicity tests should be cross-validated in multiple laboratories; reliable experimental data are necessary for the successful development of new QNAR, which requires researchers to work together with the QSAR community to provide reliable and useful data to better understand the toxicity mechanism of metal oxide nanomaterials. Third, most of the existing metal oxide QNAR models are based on limited data, which may cause some statistical errors; we should extend the types of metal oxide nanomaterials studied to reduce these errors. Meanwhile validation is vital to ensure that the predictive ability of the model is not caused by chance factors, but there are no standardized validation metrics [28]. On the other side, we use QNAR models to design safer metal oxide nanomaterials based on their structural descriptors, but some of the structural descriptors, such as constitutional structural descriptors, are not associated with characteristics of metal oxide nanomaterials, so it is necessary to select structural descriptors which are directly relate to the characteristics of metal oxide nanomaterials. Additionally, the issue of chemical doping which is reported as a potential factor causing toxicity, should also be considered in future QNAR studies. Only reliable and meaningful QNAR models can be applied to the high-throughput screening of the toxicity of metal oxide nanomaterials and to guide the production of metal oxide nanomaterials.
